# Assessment of Mycotoxin Exposure in a Rural County of Chile by Urinary Biomarker Determination

**DOI:** 10.3390/toxins13070439

**Published:** 2021-06-25

**Authors:** Claudia Foerster, Gisela Ríos-Gajardo, Patricia Gómez, Katherine Muñoz, Sandra Cortés, Carlos Maldonado, Catterina Ferreccio

**Affiliations:** 1 Institute of Agri-Food, Animal and Environmental Sciences (ICA3), University of O’Higgins, San Fernando 3070000, Chile; carlos.maldonado@uoh.cl; 2 Department of Food Science and Technology, Faculty of Pharmacy, University of Concepción, Concepción 4030000, Chile; grios@udec.cl (G.R.-G.); patriciagomezp@gmail.com (P.G.); 3 iES Landau, Institute for Environmental Sciences, University of Koblenz-Landau, 76829 Landau, Germany; munoz@uni-landau.de; 4 Centro de Desarrollo Urbano Sustentable (CEDEUS), Santiago 8320000, Chile; scortesn@uc.cl; 5 Advanced Center for Chronic Diseases (ACCDiS), Escuela de Medicina, Pontificia Universidad Católica de Chile, Santiago 8320000, Chile; cferrec@med.puc.cl

**Keywords:** aflatoxin, ochratoxin A, deoxynivalenol, zearalenone, urine biomonitoring, food, occupation, Chile

## Abstract

Aflatoxin B1 (AFB1), ochratoxin A (OTA), zearalenone (ZEN), and deoxynivalenol (DON) are frequent mycotoxins that may cause carcinogenic, mutagenic, estrogenic, or gastrointestinal effects. The aim of this study was to assess the exposure to and risk from AFB1, OTA, ZEN, and DON in 172 participants of the Maule Cohort (MAUCO) by a biomarker analysis in urine and to associate their exposure with food consumption and occupation. Mycotoxins in the first morning urine were analyzed by solid-phase extraction and quantified by Ultra-High-Performance Liquid Chromatography with a mass–mass detector. Participants’ information regarding food consumption, occupation, and other characteristics was obtained from a baseline and 2-year follow-up survey of the cohort. The prevalence and mean levels of mycotoxins in the urine were as follows: DON 63%, 60.7 (±78.7) ng/mL; AFB1 8%, 0.3 (±0.3) ng/mL; α-zearalenol (α-ZEL) 4.1%, 41.8 (±115) ng/mL; β-ZEL 3.5%, 17.4 (±16.1) ng/mL; AFM1 2%, 1.8 (±1.0) ng/mL; OTA 0.6% (1/172), 1.3 ng/mL; and ZEN 0.6%, 1.1 ng/mL. These results were translated into exposures of DON, ZEN, and aflatoxins of public health concern. Participants who consumed coffee and pepper the day before had a significantly greater presence of DON (OR: 2.3, CI95 1.17–4.96) and total ZEL (OR: 14.7, CI95 3.1–81.0), respectively, in their urine. Additionally, we observed associations between the habitual consumption of beer and DON (OR: 2.89, CI95 1.39–6.42). Regarding the levels of mycotoxins and the amount of food consumed, we found correlations between DON and nuts (*p* = 0.003), total ZEL and cereals (*p* = 0.01), and aflatoxins with capsicum powder (*p* = 0.03) and walnuts (*p* = 0.03). Occupation did not show an association with the presence of mycotoxins in urine.

## 1. Introduction

Mycotoxins are toxic metabolites produced naturally by some species of filamentous fungi, such as *Aspergillus, Fusarium*, and *Penicillium* [1,2]. Fungal growth can occur before or after harvest, during storage, or in foods, especially in environments with high humidity and temperatures, followed by mycotoxin production. Most mycotoxins are chemically stable and persist after food processing [3,4]. The most investigated mycotoxins are aflatoxin B1 (AFB1), ochratoxin A (OTA), zearalenone (ZEN), and deoxynivalenol (DON), causing carcinogenic, mutagenic, estrogenic, and gastrointestinal effects in humans and animals [5]. Human exposure to these mycotoxins occurs predominantly through the consumption of contaminated foods [6]. Daily exposure to mycotoxins has been measured in an indirect way by an estimated daily intake (EDI), which is based on consumption and the mycotoxins’ concentrations in foods [7], as well as by a direct exposure assessment utilizing a probable daily intake (PDI); the latter is based on biomarker measurements in biological fluids, such as urine and blood, and the excretion rate of the mycotoxins [8]. This method is the most accurate and has been used to estimate individual mycotoxin intakes, including all sources of exposure [9]. Urine is usually preferred for population-based studies because it is a noninvasive method; its limitations are the daily variations in urine composition and that the mycotoxin excretion rates varies among individuals [6,10]. Urinary biomarkers are better indicators for short-term variations in exposure, as blood biomarkers may not reflect this because of the protein-binding properties of some mycotoxins (e.g., aflatoxin and OTA) [8,11].

The main metabolites of mycotoxins found in urine are aflatoxin M1 (AFM1), a product of the hydroxylation of AFB1 in the liver [11]; ochratoxin α (OTα), the hydrolyzed form of OTA [12]; α-zearalenol (α-ZEL) and β-zearalenol (β-ZEL), which are products of the Phase II biotransformation of ZEN [13]; and de-epoxy-deoxynivalenol (DOM-1) and deoxynivalenol-glucuronide (DON-GlcA), which results from the biotransformation of DON [14]. However, aglycone mycotoxins are also usually found in urine and often complementarily used in biomonitoring studies [6,8,15,16].

After exposure estimation, further health risk assessment is usually conducted in terms of the tolerable daily intake (TDI) or a Margin of Exposure (MoE) approach. For the risk characterization of non-carcinogenic toxins, a Hazard Quotient (HQ) is usually assessed, being the ratio between the calculated PDI and the reference TDI determined by toxicological studies in sensitive laboratory species [17]. The MoE is the ratio of the benchmark dose lower bound (BMDL) of a dose–response curve, usually at the 10% effect (BMDL10), and the estimated PDI [18]. The MoE approach is usually used for carcinogenic toxins, such as aflatoxin. Recent studies regarding the uncertainty of OTA and kidney carcinogenicity estimated that the previous tolerable weekly intake (TWI) of 120 ng/kg body weight (bw) was no longer valid and an MoE approach needs to be applied for risk characterization [19].

In Chile, previous research showed that OTA and aflatoxins were the most prevalent mycotoxins in food, especially from imported spices and capsicum (chili and paprika powder). An indirect exposure assessment based on these data estimated that aflatoxin contamination of cereals, dairy, and nuts should be considered a health concern [20]. Additionally, population-based studies showed OTA in plasma, urine, and breast milk [21,22,23], and aflatoxin in plasma [24]. The latter case–control study reported aflatoxin–albumin adducts in serum associated with the risk of gallbladder cancer (GBC) (Odds Ratio (OR): 13.2; 95% Confidence Interval (CI95) 4.3–47.9), and higher consumption of capsicum among GBC patients than among the controls (OR: 13.2; CI95 4.3–47.9). The higher GBC risk associated with capsicum consumption had been previously noted by Serra et al. [25]. Thus, it has been proposed that capsicum contaminated with aflatoxin could in part explain the high rates of GBC in the Chilean population, with it being especially high in areas consuming high quantities of capsicum [26].

Even though aflatoxins and OTA are the most sampled and prevalent mycotoxins in food in Chile [20], there is no current information regarding the direct estimation of exposure based on measurements of different mycotoxins in the population in the form of biomonitoring studies. It is hypothesized that rural populations may experience higher mycotoxin exposure than urban populations, due to occupational exposures [27]. Currently, exposures in Chile are not associated with specific foods or occupations. Thus, the aim of this study was to assess exposure to AFB1, OTA, ZEN, and DON in residents of an agricultural county with high rurality by measuring the mycotoxin biomarkers in urine, and to explore the mycotoxins’ associations with specific food items and occupational exposures. For this aim, we nested our study in the agricultural county of Molina, in the population-based Maule Cohort, MAUCO [28,29].

## 2. Results

### 2.1. Description of the Population

The description of the participants is shown in Table 1. The 172 participants had an average age of 57 (±9.3) years, and no differences were observed between the sexes in age, education, ethnicity, and current habits regarding smoking, drinking alcohol, and physical activity. The only significant difference was the higher number of men in agricultural work (*p* = 0.001).

### 2.2. Food Consumption Reported by the Participants

The prevalence of the selected food items previously associated with mycotoxins was obtained. It is expressed as the percentage of participants who reported themselves consuming it the day before their urine sample. In order of frequency of consumption, the following percentages were obtained: capsicum powder (99%), tea (81%), meat (64%), dairy (46%), coffee (30%), rice (29%), legumes (20%), cereals (11%), peanuts (10%), maize and wine (7%), and walnuts (6%). Other nuts, coffee, and spices had a prevalence of less than 4%. In the case of habitual consumption, the prevalence from the most consumed was legumes (93%), dairy (81%), nuts (70%), wine (51%), maize (32%), capsicum (32%), beer (28%), whole grain cereal (27%), fresh green chili (29%), and fresh red chili (21%).

Consumption in grams per day (g/day), resulting in habitual consumption and the 24 h recall reported by the participants, is shown in Table 2. We did not find differences between the sexes regarding food consumption, with the exceptions of a higher beer consumption among men and a higher ginger consumption among women (Table 2).

### 2.3. Occurrence and Concentration of Mycotoxins in Urine

The prevalence and mean (standard deviation (SD)) concentration of mycotoxins in urine, along with the limits of detection (LOD) and quantification (LOQ) of the method, are shown in Table 3. The analysis of urine samples showed that DON was the most frequently occurring mycotoxin (73%), with 63% of the samples above the LOQ of the method. It was followed by AFB1 (8%), α-ZEL (8%), and β-ZEL (7.5%). The higher concentrations creatinine-adjusted were for DON with 64.6 (SD: 205.8) ng/mg creat, β-ZEL with 21.9 (SD: 57.8) ng/mg creat, α-ZEL with 19.1 (SD: 25.2) ng/mg creat, and much lower for AFB1 with 0.3 (SD: 0.2) ng/mg creat. The metabolites OTα and DOM-1 were not found in urine. We did not find difference between the sexes in relation to mycotoxin prevalence or levels. The age groups between 45–54 years and 65–75 years had significantly more DON than the 35–44 and 55–64 age groups (*p* = 0.03). Moreover, ten subjects had a co-occurrence between aflatoxins and DON in their urine; ten subjects had ZEN metabolites, along with DON; and one participant showed the presence of DON, aflatoxins, and ZEL.

### 2.4. Association Between Food Consumption and Mycotoxins in Urine

Considering food items of special interest, we found that participants who consumed coffee and pepper the day before had a significantly greater presence of DON (OR: 2.3, CI95 1.17–4.96) and total ZEL (OR: 14.7, CI95 3.1–81.0), respectively, in their urine. Furthermore, a protective association between dairy and DON (OR: 0.42, CI95 0.23–0.75) was observed. Regarding habitual consumption of the selected food items, we observed a protective association between nuts and DON (OR: 0.37, CI95 0.15–0.83), as well as between wine and total ZEL (OR: 0.29, CI95 0.11–0.69). Additionally, borderline associations between the habitual consumption of beer and DON (OR: 1.99, CI95 1.02–4.13) as well as coffee and aflatoxins (OR: 2.52, CI95 1.04–6.12) were observed. Crude ORs for all the selected food items are shown in Supplementary Table S1. These associations remained after adjusting for sex and age. In the case of the association between beer and DON, the adjusted ORs were significant (OR: 2.89, CI95 1.39–6.42), and risk was associated with men and younger participants (<54 years old).

Regarding the levels of DON, aflatoxins, and ZEN metabolites and the amount (g/day) of food consumed, regression models revealed significant correlations between DON and nuts (R^2^: 0.07, *p* = 0.003), total ZEL and cereals (R^2^: 0.04, *p* = 0.01), as well as between aflatoxins with capsicum powder (R^2^: 0.18, *p* = 0.03) and walnuts (R^2^: 0.44, *p* = 0.03). Regressions of the selected food items are shown in Supplementary Table S2.

### 2.5. Association Between Occupation and Mycotoxins in Urine

No significant associations between food handling or agricultural work and the presence of mycotoxins in urine were found. Of the 19 food handlers, only two were mill workers or bakers, and both had DON in their urine, with levels of 30 and 66 ng/mg, adjusted for creatinine. We found 11 participants with very high DON levels (>100 ng/mg creatinine-adjusted). Among them, nine (82%) were women, and they differed in neither food consumption nor occupation.

### 2.6. Dietary Exposure and Risk Assessment

**Figure 1:**
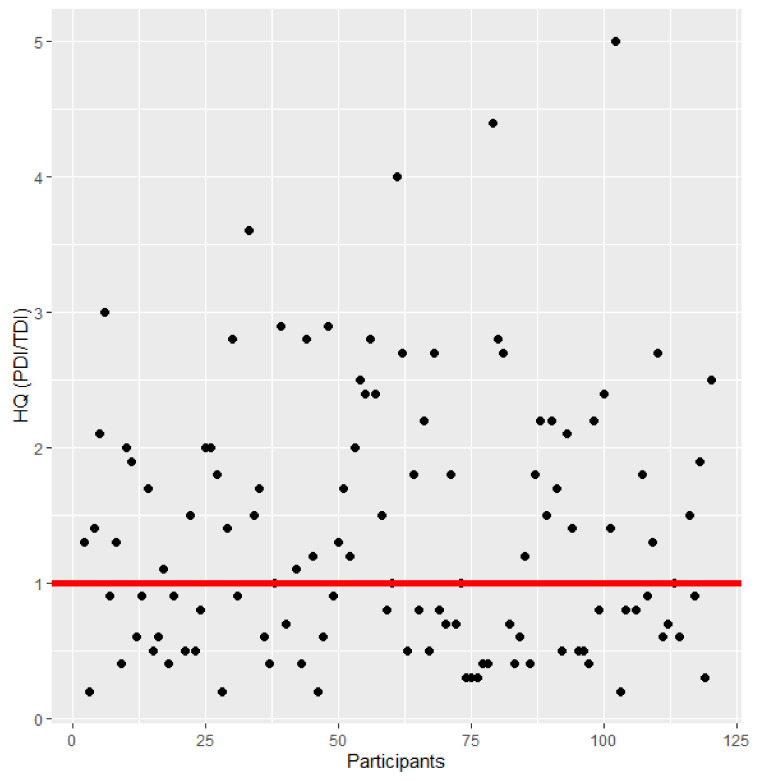
Hazard Quotient (HQ) of prevalent participants for deoxynivalenol (DON) in urine (*n* = 125). The red line shows an HQ of 1. If the HQ > 1, the exposure could be of health concern. An HQ > 5 (outliers) was not considered in the figure (*n* = 6).

Exposure was calculated in prevalent participants using probable daily intake (PDI). The mean (SD) PDI for DON was 2532 (6921) ng/kg bw creatinine-adjusted; a mean of 5997 (9556) ng/kg bw creatinine-adjusted was estimated for ZEN; and a mean of 1.1 (2.3) ng/kg bw creatinine-adjusted was calculated for AFB1. Compared to the tolerable daily intake (TDI) of DON (1 µg/kg bw/day), the exposure of 55% of the participants (66/125) resulted in a public health concern (Figure 1). A woman of 62 years old had a PDI as high as 68,860 ng/kg bw creatinine-adjusted.

Regarding ZEN, when compared to the TDI (0.25 µg/kg bw/day), as well as aflatoxins compared to the Margin of Exposure (MoE) for carcinogenic effects (0.4 µg/kg bw per day), 100% of the participants tested positive for zearalenone metabolites (18/18) and for aflatoxins (16/16) in their urine, representing a potential health concern in terms of exposure (for more details, see Supplementary Material Table S3). Two women had a PDI for ZEN above 40,000 ng/kg bw creatinine-adjusted.

## 3. Discussion

High prevalence and concentrations of DON, low prevalence and high concentrations of ZEN metabolites, and low prevalence and concentrations of aflatoxin were observed. Compared with other similar studies, while prevalence was lower than reported in Europe [30,31,32,33,34], Brazil [35], USA [36], Haiti [32], and South Africa [37], levels in Chile were higher than reported for DON and ZEN metabolites (Table 4). A study of pregnant women in Croatia found high levels of DON and its conjugates DON-3-GlcA and DON-15-GlcA [38], reporting similar total DON levels than our study. In our case, the samples were hydrolyzed, which means that the DON aglycone and DON conjugate (glucuronide or sulfate) are being measured simultaneously.

The MAUCO participants were residents of the Molina county, in Central Chile, an area characterized by a temperate climate, with a high range of variation in temperature and rainfall regimes [39]; this region is especially prone to *Fusarium* development and the production of zearalenone, fumonisins, and trichothecenes, such us DON [40]. A food item that was not considered in the food surveys because of its universal consumption was bread. According to the National Food Consumption Survey (ENCA), 99.1% (CI95 98.8–99.4%) of the Chilean population eats bread [41], with a median consumption of 151.9 (85.2–233.7) g/day [41]. The bread consumed in Chile is mainly wheat-based, with 63% local production [42]. During the summer of 2017 (the year of the urine sampling), Chile had its second hottest summer in more than 50 years. The Annual State of the Climate Report of the USA [43], highlighted the unusual weather conditions in the country, with extended periods of drought and extreme heatwaves, leading to increased rainfall and the heaviest snowfall in nearly 100 years. Curicó, a city near Molina, also broke its record for maximum temperature. These exceptional conditions may be produced by local outbreaks of *Fusarium* and could explain the high levels of DON observed in this study, as the accumulation of trichothecene mycotoxins in the kernels are strongly weather dependent [44].

The DON levels observed in this biomonitoring do not correlate with the levels reported by the Chilean Mycotoxin Surveillance Program, where all samples analyzed for DON (mainly wheat flour) were below the regulation [20]. This means that sampling programs must be encouraged to identify the dietary contribution of DON. In this regard, significant associations were observed between beer consumption and DON. Beer is often found to have been contaminated with DON, ZEN, and other mycotoxins prevalent in cereals [45,46,47]. There has been an important and sustained increase in the consumption of beer in Chile in recent years, where consumption has gone from 25 to 44 L/year per capita [48]. These results suggest that beer must be incorporated into the Chilean Mycotoxin Surveillance Program, and further analysis is needed in this matrix. Coffee consumption was also associated with DON prevalence. This association can be explained because a large percentage of coffee consumed in Chile is instant (95%) [49], of which an unknown percentage may be cereal coffee, such as barley coffee. Currently, coffee is analyzed only for OTA [20], so exploring other mycotoxins in this food item is necessary. Although *Fusarium* toxins have been found in nuts [50,51], more research is needed to explain the correlation between the DON levels and nuts consumption regarding possible cross-contamination between grains or if they are consumed with cereal-based foods. On the other hand, breakfast cereal consumption, which could also explain these high levels, is lower than 27% in adults according to ENCA [41], which is in accordance with the consumption reported in this study. Another possible explanation of the high DON levels seen in this study is imported processed products based on cereals that were not detected by the Surveillance Program due to the low number of analyses made for this mycotoxin (approx. 25 analyses per year) [20].

We explored both 24 h recall and habitual consumption using a validated Mediterranean diet survey in food items associated with mycotoxin exposure. The association between capsicum powder and aflatoxin was expected as it is the most mycotoxin-contaminated food item in Chile [20]. Capsicum is prone to aflatoxin contamination [52,53,54], but is not usually associated with aflatoxin exposure [15]. Other food items found to be associated with mycotoxins in urine were walnuts and aflatoxins, while ZEN was associated with cereals; these are both associations found in previous studies [15,55,56,57]. However, due to the low number of positive samples, correlations made for aflatoxins and ZEL may be not predictive.

Regarding the potential health effects of DON in this population, acute effects, such as gastroenteritis, and chronic health effects, such as altered nutritional efficiency, weight loss, and anorexia [15], must be studied given the exceptionally high exposures estimated. Furthermore, ZEN exposures should be continually monitored in this population because of their estrogenic effects [5,58]. For assessing chronic effects, prospective studies regarding the possible association between mycotoxins and chronic digestive diseases in MAUCO must be designed [59]. The Maule region has one of the highest cancer mortality rates in Chile, especially when it comes to digestive cancers, such as gastric, gallbladder, and esophagi cancer [60].

We did not find an association between occupation and mycotoxins in urine. This could be explained because most agricultural participants reported their work in open environments (fruit, cereals, and vegetable harvest), whereas occupational exposure have been mainly associated with airborne mycotoxin due to poor ventilation and inappropriate protective clothing [61,62,63]. In the case of the 19 food handlers of this study, the majority worked as cooks in kitchens or in delivery roles. Only two of them worked in a bakery or mill, and both presented DON in their urine. In this regard, future studies must be focused on mill and bakery workers to assess occupational exposure.

Although this study presents new information about mycotoxin exposure, the results must be interpreted with caution as they represent only a small part of the population. Compared to the MAUCO participants [28], even though there were no differences between the proportion of men and women between the two studies, in our sample, women were significantly older than the MAUCO population. This could limit the representativeness of the results. Another limitation was that dietary intake and other descriptive information of the participants were self-reported, so misreporting could not be excluded. Additionally, habitual consumption was obtained from a Mediterranean diet survey, which did not have all of the most mycotoxin-prevalent foods. Due to the long half-lives of aflatoxins and OTA, urine biomarkers of these mycotoxins (especially for once-off urine samples, such as in this study) may not be the most effective methods of choice [10,15]. Furthermore, AFM1, the main biomarker of AFB1, is excreted within the first 1–4 days, and unmetabolized aflatoxin B1 is excreted in the first day [61]. This fact could explain the higher prevalence of AFB1 in this sample. For further and more accurate analyses, investigating biomarkers such as aflatoxin–albumin adduct and OTA in serum should be considered [6,8]. Additionally, the LODs of AFM1 and OTA of this method were higher than usually detected, so the results may be underestimated.

Despite these limitations, this is the first study to report the simultaneous presence of aflatoxins, OTA, DON, and ZEN in the urine of members of the general Chilean population. As such, this study has presented new information regarding the associations between direct mycotoxin exposure and food.

## 4. Conclusions

This study presents new information about mycotoxin exposure in Chile. High prevalence and concentrations of DON, low prevalence and high concentrations of ZEN metabolites, and low prevalence and concentrations of aflatoxin were observed in the urine of 172 participants. The risk assessment estimations based on those levels were translated into a high exposure risk for DON, AFB1, and ZEN in the participants. The significant associations observed between mycotoxins and food consumption were the following: DON with nuts, coffee, and beer; ZEN metabolites with pepper and cereals; and aflatoxins with capsicum powder and walnuts.

Further studies must address the following: (i) continuous biomonitoring of these mycotoxins to assess if these levels were due to climate exception or they are habitual regarding our unique consumption patterns; (ii) prospective population-based studies for assessing the health effects of the high exposures observed in this study; (iii) sampling and analysis of food items not usually considered in the surveillance program, e.g., beer and bread; and (iv) assessments of occupational exposure, especially in relation to mill workers and bakers.

## 5. Materials and Methods

### 5.1. Study Population

The study subjects were a sample (*n* = 172) of the Maule Cohort (MAUCO), in Molina, Region del Maule, Chile (latitude S35°21’12.13” and longitude O70°54’34.34”). A priori power analyses indicated that 160 subjects of the 8000 MAUCO participants at the time would be sufficient for significant results, albeit with the assumption of 90% prevalence (as in the case of OTA in [21,22,23]), with 80% power and 95% confidence. Participants were selected by a convenience sampling, which included around 50% agricultural workers and all participants who were working in food handling that had started their 2-year follow-up of MAUCO in 2017.

MAUCO is the first prospective population-based cohort of cardiovascular disease and cancer in Chile [28,29]. All the residents of the Molina County aged 38 to 74 years who were able to autonomously consent to join the cohort and who did not have a late-stage disease were eligible to enter the cohort. Enrollment was initiated in January 2015, and participants will be followed for at least 10 years. The methods and baseline findings have been reported elsewhere [28,29]. According to Berdegué et al. [62], Molina belongs to Group 2 of the rural counties, a group that represents 44% of the rural population of Chile.

### 5.2. Diet and Occupation Assessment

In 2015, MAUCO’s participants answered a habitual Mediterranean diet consumption poll as part of the health and lifestyle questionnaire. The question for each specific food was as follows: “On average, in the last 12 months, how many servings of did you consume per week?”. Possible answers were alternatives: no consumption, less than a portion, 1 portion, or 2 or more portions. A sample of the first morning urine of the participants was taken as part of their 2-year follow-up (from May to November 2017), and was frozen until analysis. A food questionnaire on dietary intake the previous day was administered the day of the urine sample, along with their current job. Food consumption was assessed from yes/no consumption and the quantity of the consumption in g per day. A serving was defined depending on the type of food, i.e., a cup of cereals (33 g); a cup of dairy (200 mL); a teaspoonful of coffee, tea, spices (5 g); a cup of maize (100 g); a plate of legumes (190 g); a portion of meat (85 g); and a handful of peanuts or mix of nuts (30 g), including walnuts, almonds, hazelnuts, cashews, pistachios, and peanuts.

For occupation, the participants were asked the following: “Do you have a current job? What kind of activity does the company, business, industry, service, or office where you work do? Does your current job consist in the production, processing, or handling of food (for human and animal consumption)?”.

### 5.3. Reagents and Chemicals

Standards of AFM1, AFB1, DON, DOM-1, OTA, ZEN, α-ZEL, and β-ZEL were purchased from Sigma-Aldrich (St. Louis, MO, USA). OTα was purchased from Romer Labs Diagnostic (Tulln, Austria). Internal standards U-(13C17) aflatoxin B1 (0.5 µg/mL) in ACN, U-(13C17)-aflatoxin M1 (0.5 µg/mL) in ACN, and U-(13C20)-ochratoxin A (10 mg/mL) U-(13C15)-deoxynivalenol (25 mg/mL) were purchased from Romer Labs Diagnostic. Acetonitrile (ACN), methanol (LC–MS grade), and acetic acid (96%) were purchased from Merck (Darmstadt, Germany). Formic acid and ammonium acetate were obtained from Sigma-Aldrich. The glucuronidase/arylsulfatase enzyme from *Helix pomatia*, respectively 30 U/mL and 60 U/mL units, was purchased from Sigma-Aldrich. OASIS^®^ HLB columns, 60 mg, 1 cc were purchased from Waters (Milford, MA, USA).

### 5.4. Urine Sample Extraction

The first morning urine samples were unfrozen and centrifuged at environmental temperature for 3–5 min at 5600× *g* prior to extraction. Then, the urine concentration was calculated by the creatinine percentage by a creatinine kit (Creatinine Respons KIT, Sigma-Aldrich) and further measured by spectrophotometry. Three milliliters of urine were mixed with 250 µL of sodium acetate buffer (1.4 M) at pH 5.0 and 40 µL of β-glucuronidase/arylsulfatase enzyme (Sigma-Aldrich), and was in-move incubated for 16 h in an oven at 37 °C. Oasis HLB Prime columns 1 cc (Waters) were conditioned with 100% methanol and then distilled water (Merck). The hydrolysate was later passed through them. The columns were cleaned twice with distilled water, and the contents were subsequently eluted with 3 mL of acetonitrile 100%. The eluate was evaporated under a gentle nitrogen stream (Thermo Scientific, MA, USA) at 45 °C and then reconstituted with 450 µL of acetonitrile and subsequently was filtered with a 0.22 µm Teflon syringe filter. The filtrate was received in amber vials and [13C]-labelled internal standards were added for further quantification by Ultra-High-Performance Liquid Chromatography with a mass spectrometric detection detector (UPLC-MS/MS; Shimadzu, Kyoto, Japan).

### 5.5. Chromatographic Conditions

The LC-MS analysis was performed with a Shimadzu (Kyoto, Japan) Nexera X2 UHPLC system, which consisted of a LC-30AD pump, a DGU-20A5R degassing unit, an SIL-30AC autosampler, a CTO-20AC column oven, a CBM-20A communication module, an SPD-M20A diode array detector, and an ESI-LCMS-8030 triple quadrupole mass spectrometer. The system was controlled by LabSolution 5.8 software. The separation was achieved by a Phenomenex (Torrance, CA, USA) Kinetex XB-C18 column (100 mm × 4.6 mm, 2.6 μm), with an oven temperature of 30 °C and a flow rate of 0.4 mL/min. The mobile phase A was 0.1% acetic acid in Milli-Q water, and phase B was acetic acid 0.1% in acetonitrile. The volume of injection was 20 µL. The gradient used started with 10% B for 5 min, and then increased to 50% B over 3 min. Then, eluent B was raised to 95% until min 15.0 followed by a hold-time of 2.0 min and subsequent 3 min column re-equilibration at 10% B. The triple quadrupole mass spectrometer had an electrospray ionization source (ESI). In general, the detection parameters were the following: collision gas argon, nebulizer gas (N2) of 3 L/min, desolvation gas (N2) of 15 L/min, desolvation line temperature of 250 °C, and heat block temperature of 400 °C. Full-scan spectra were acquired from *m/z* 100 to 2000 with a multiple reaction monitoring working mode. The UPLC-MS/MS parameters for the detection of the targeted mycotoxins are shown in Supplementary Table S4a. Quantification was done by interpolation of the data in the calibration curve for all mycotoxins. Matrix compensation was done via IS. The lowest detection levels for the investigated mycotoxins were set as the lowest level of the calibration curve (Table S4b). The recovery of the method is specified in Table S4c.

### 5.6. Exposure Assessment

The probable daily intake (PDI) of mycotoxin was calculated according to Equation (1):

PDI (ng/kg bw/day) = (C (ng/mL) × V (mL) × 100)/(BW (kg) × E (%)) (1)

where C is the urinary concentration of the mycotoxin biomarker (ng/mL), V is the mean volume of daily urine production in adults (1500 mL), BW is the individual weight of the participants (kg), and E is the mean urinary excretion rate per mycotoxin (%): 1.5% for aflatoxins [63], 2.5% for OTA [64], 9.4% for ZEN, and 72% for DON [65]. Concentrations were assumed to be the mean value between LOD and LOQ in <LOQ.

PDI was also calculated based on the creatinine-adjusted biomarker concentrations (ng/mg of creatinine). Samples too diluted (creatinine <0.3 mg/mL) or too concentrated (creatinine >3 mg/mL) were excluded from the estimations [66].

### 5.7. Risk Characterization

For aflatoxins, the Margin of Exposure (MoE) was calculated as the ratio between the reference benchmark dose level (BMDL) at 10% (BMDL10) and the estimated PDI. According to the CONTAM Panel of EFSA, we used a BMDL10 of 0.4 µg/kg bw per day for the incidence of HCC; a calculated MOE below 10,000 implies a high health concern [67]. For DON and ZEN risk characterization, a Hazard Quotient (HQ) was assessed, being the ratio between the calculated PDI and the reference tolerable daily intake (TDI) determined by toxicological studies in sensitive laboratory species [17]. A TDI of 1 μg/kg bw per day was used for DON [68] and a TDI of 0.25 μg/kg bw was used for ZEN [69]. A HQ < 1 was considered a health concern.

### 5.8. Statistical Analysis

A descriptive analysis of the available data was performed using the software R-project version 4.0.2 (https://www.r-project.org). Differences between the sexes for the sociodemographic characteristics were analyzed using the Student’s t-test (t.test) and Kruskal–Wallis test (kruskal.test) for continuous variables, while categorical variables were analyzed with a Chi-squared test (chisq.test). To assess possible associations between the prevalence of mycotoxin levels and food consumption patterns, linear regression was used. On the other hand, a generalized linear model was used to fit the presence of mycotoxins and food consumption, assuming a binomial distribution of the explanatory variable and a logit link function. The *p*-value in food consumption in the total population, stratified by sex, was calculated using the kruskal.test R function, since data were not normally distributed. A *p*-value <0.05 was considered significant.

## Figures and Tables

**Table 1:** Sociodemographic characteristics of participants of the study (*n* = 172), stratified by sex.

Characteristics	All	Women	Men	*p*-Value Sex Difference
	*n* = 172	*n* = 81	*n* = 91	
**Age, mean ± SD (years old)**	57.4 ± 9.3	58.5 ± 8.8	56.4 ± 9.7	0.129 ^a^
35–44	10.5	6.2	14.3	
45–54	26.7	27.2	26.4	
55–64	39.0	40.7	37.4	
65–74	23.8	25.9	22.0	
**Body mass index (BMI)**	29.3 ± 4.5	29.5 ± 5.3	29.1 ± 3.8	0.536 ^a^
**Self-reported ethnicity (%)**				0.222 ^b^
Chilean/Hispanic	98.8	97.5	100	
Mapuche	1.2	2.5	0	
**Schooling (years)**				0.177 ^b^
≤8	55.8	61.7	50.5	
9–12	34.3	27.2	40.7	
≥13	9.9	11.1	8.8	
**Current smoking (%)**	43.9	51.1	37.7	0.269 ^b^
**Current alcohol drinking (%)**	66.7	60.5	72.2	0.143 ^b^
**Current physical activity (%)**	22.5	17.3	27.1	0.183 ^b^
**Agriculture worker (%)**	48.2	34.2	60.0	**0.001 ^b^**
**Food handling worker (%)**	11.4	7.8	14.4	0.226 ^b^

^a^*p*-values obtained from t-tests (continuous variable); ^b^*p*-values obtained from Chi-square tests (categorical variables).

**Table 2:** Daily food consumption (g or mL/day) for the participants of the study (*n* = 172), stratified by sex.

Consumption	Food Item	All (Mean ± SD)	Women (Mean ± SD)	Man (Mean ± SD)	*p*-Value
**Habitual consumption** **(g or mL/day)**	Capsicum	0.8 ± 2.0	0.7 ± 1.9	1.0 ± 2.0	0.230
	Cereal (whole)	6.0 ± 13.1	8.2 ± 16.6	4.1 ± 8.5	0.540
	Beer	62.6 ± 268.2	45.6 ± 350	77.8 ± 165.2	0.002
	Maize	149.2 ± 166.5	125 ± 84.7	165.7 ± 204.3	0.898
	Nuts	9.8 ± 9.5	10 ± 9.9	9.5 ± 9.1	0.988
	Dairy	110.6 ± 95.8	117.4 ± 96.8	104.5 ± 95.1	0.338
	Legumes	53.4 ± 31.9	50.1 ± 28.8	56.4 ± 34.4	0.274
	Wine	134.9 ± 421.1	89.1 ± 309.7	175.6 ± 498.1	0.103
**24 h consumption** **(g or mL/day)**	Capsicum	0.7 ± 1.9	0.8 ± 2.1	0.7 ± 1.6	0.863
	Coffee	1.9 ± 3.7	1.6 ± 2.9	2.2 ± 4.3	0.408
	Meat	60.4 ± 56.8	57.5 ± 55.9	63 ± 57.8	0.448
	Cereal	3.5 ± 10	3.9 ± 10.5	3.1 ± 9.5	0.607
	Maize	4.8 ± 19.7	5.2 ± 20.4	4.4 ± 19.2	0.623
	Ginger	0.2 ± 1.1	0.4 ± 1.4	0.1 ± 0.5	0.019
	Legumes	45.3 ± 99.9	44.6 ± 91.5	45.9 ± 107.3	0.767
	Peanut	3.0 ± 9.0	3.0 ± 9.0	3.0 ± 9.0	0.998
	Walnut	1.3 ± 5.9	1.3 ± 4.9	1.2 ± 6.7	0.595
	Tea	7.6 ± 6.5	8.1 ± 7.4	7.0 ± 5.5	0.551

SD: Standard deviation.

**Table 3:** Prevalence and concentrations of detected mycotoxins found in the urine of the 172 participants of this study, along with the limit of detection (LOD) and quantification (LOQ) of the method.

Mycotoxin	LOD (ng/mL)	LOQ (ng/mL)	Over LOQ (%)	Prevalence (%)	Mean (SD) (ng/mL)	Mean (SD) ng/mg Creat ^a^	Median (IQ Range) (ng/mL)
AFB1	0.08	0.1	7	8	0.3 (0.3)	0.3 (0.2)	0.3 (0.1–0.3)
AFM1	0.8	1.1	1	1	1.8 (1.0)	4.3 (2.8)	1.8 (1.5–2.2)
OTA	0.4	2.1	0	0.6	-	-	-
DON	6.6	20.1	63	73	60.7 (78.7)	64.6 (205.8)	37.6 (23.2–61.1)
ZEN	0.5	1.7	0	0.6	-	-	-
α-ZEL	1.2	3.7	6	8	41.8 (115.5)	19.1 (25.2)	11.8 (6.2–16.5)
β-ZEL	0.7	2.3	7.5	7.5	17.4 (16.1)	21.9 (57.8)	8.6 (6.9–31.3)

^a^ ng/mg creatinine-adjusted; IQ range: interquantile range; SD: standard deviation.

**Table 4:** Comparative prevalence (%) and mean levels (ng/L) of aflatoxins, ochratoxin A (OTA), deoxynivalenol (DON), and zearalenone (ZEN), as well as their metabolites α-zearalenol (α-ZEL) and β-zearalenol (β-ZEL) analyzed in adults’ urine of different countries.

	Mycotoxin	Chile (This study)	Belgium [30]	Italy [31]	Germany [32]	Germany [33]	Sweden [34]	Brazil [35]	Haiti [32]	USA [36]	South Africa [37]
**Mycotoxin Prevalence (%)**	**Aflatoxins**	9	-	6	0	-	-	12	11	13	0
	**OTA**	0.6	35	100	15	77	51	27	47	87	96
	**DON**	55	37	96	8	100	63	88	24	-	87
	**ZEN**	0.6	-	100	-	100	37	7	-	-	100
	**α-ZEL**	8	0.4	100	0	46	21	0	4	-	92
	**β-ZEL**	6	-	98	-	23	18	0	-	-	75
**Mycotoxin Mean Level (ng/L)**	**Aflatoxins**	500	-	68	-	-	-	20 *	60	4670	-
	**OTA**	-	27.8	140	97	66	460	20 *	110	6200	24
	**DON**	60,700	3900	11,890	2000	6850	3370	12,000 *	3200	-	4940
	**ZEN**	1100	-	60	-	31	30	20 *	-	-	204
	**α-ZEL**	41,800	5000	80	-	16	30	-	1460	-	247
	**β-ZEL**	17400	-	90	1420	8	20	-	-	-	244

* Creatinine-adjusted.

## Data Availability

Additional information is in Supplementary Material.

## Supplementary Materials

The following are available online at https://www.mdpi.com/article/10.3390/toxins13070439/s1, Table S1. Odds ratio (OR) and Confidence Intervals (CI) between the selected food items and the presence of mycotoxins in the urine of the *n* = 172 participants; Table S2. Calculated regressions of the levels of mycotoxins in urine found and the grams per day consumed by the 172 participants of the study.; Table S3. Levels in ng/kg bw creatinine-adjusted, probable daily intake (PDI), and risk estimation by the Hazard Quotient (HQ) or Margin of Exposure of mycotoxins in urine of the participants of the study.; Table S4. LC-MS/MS parameters, equations, and recovery for the detection of the targeted mycotoxins.
