# Supplementary material for: Assessment of Mycotoxin Exposure in a Rural County of Chile by Urinary Biomarker Determination
Source: Toxins (Basel). 2021 Jun 25;13(7):439. doi: 10.3390/toxins13070439 (PMC8309762; doi:10.3390/toxins13070439)
Supplement: Supplementary file 1 [file toxins-13-00439-s001.zip › toxins-1262768-SI.pdf]

Supplementary Materials: Assessment of Mycotoxin Exposure in a Rural County of Chile by Urinary Biomarker Determination

Claudia Foerster, Gisela Ríos-Gajardo, Patricia Gómez, Katherine Muñoz, Sandra Cortés, Carlos Maldonado and Catterina Ferreccio

Table S1. Odds ratio (OR) and Confidence Intervals (CI) between the selected food items and the presence of mycotoxins in the urine of the *n* = 172 participants. **Table S1a**, shows the association between food consumption and zearalenone metabolites (α and β zearalenol, ZEL); **Table S1b**, shows the association between food consumption and deoxynivalenol (DON); and **Table S1c**, shows the association between food consumption and aflatoxin B1 (AFB1) and aflatoxin M1 (AFM1).

Table S1. a. Association between food consumption and zearalenone metabolites (α and β zearalenol, ZEL).

	Food Item	*p*-Value	Coefficient	OR	5%	95%	RR	Total ZEL		
								**Food**	**Yes**	**No**
Habitual Consumption	Dairy	0.315	-0.619	0.538	0.206	1.614	0.583	**Yes**	14	130
								**No**	4	20
	Nuts	0.257	0.878	2.407	0.777	10.939	2.233	**Yes**	16	113
								**No**	2	34
	Whole cereal	0.623	0.262	1.300	0.518	3.057	1.261	**Yes**	6	40
								**No**	12	104
	Capsicum powder	0.391	-0.566	0.568	0.167	1.543	0.600	**Yes**	3	37
								**No**	15	105
	Legumes	0.991	16.102	9842450.491	0.000	NA	Inf	**Yes**	19	141
								**No**	0	12
	Fresh green chili	0.635	-0.262	0.770	0.291	1.832	0.793	**Yes**	5	45
								**No**	14	97
	Fresh red chili	0.952	-0.036	0.964	0.328	2.455	0.968	**Yes**	4	32
								**No**	14	108
	Maize	0.014	-2.853	0.058	0.008	0.406	0.109	**Yes**	3	52
								**No**	2	2
	Beer	0.195	-0.852	0.427	0.126	1.153	0.463	**Yes**	3	45
								**No**	15	96
	Wine	0.025	-1.235	0.291	0.110	0.694	0.332	**Yes**	5	82
								**No**	13	62
24h Recall	Capsicum powder	0.995	15.269	4277088.650	0.000	NA	Inf	**Yes**	18	147
								**No**	0	2
	Pepper (black and white)	0.005	2.688	14.700	3.102	81.049	6.480	**Yes**	3	2
								**No**	15	147
	Curcumin	0.995	-16.206	0.000	NA	INF	0.000	**Yes**	0	5
								**No**	18	144
	Ginger	0.992	-15.690	0.000	NA	INF	0.000	**Yes**	0	8
								**No**	18	142
	Dried milled maize (chuchoca)	0.997	-15.582	0.000	NA	Inf	0.000	**Yes**	0	1
								**No**	18	146
	Cooked maize (mote de maíz)	0.995	-15.262	0.000	NA	INF	0.000	**Yes**	0	2
								**No**	18	148
	Fresh maize	0.783	-0.297	0.743	0.073	3.293	0.765	**Yes**	1	11
								**No**	17	139
	Peanuts	0.883	0.118	1.125	0.241	3.664	1.110	**Yes**	2	15
								**No**	16	135
	Walnuts	0.852	-0.201	0.818	0.080	3.663	0.834	**Yes**	1	10

								**No**	17	139
	Other nuts	0.995	-16.199	0.000	NA	INF	0.000	**Yes**	0	5
								**No**	18	145
	Dried fruit (raisins, peaches, figs)	0.995	-16.402	0.000	NA	INF	0.000	**Yes**	0	6
								**No**	18	142
	Dairy	0.552	-0.304	0.738	0.309	1.688	0.762	**Yes**	7	69
								**No**	11	80
	Meat	0.449	-0.381	0.683	0.298	1.594	0.713	**Yes**	10	97
								**No**	8	53
	Salmon	0.995	-16.206	0.000	NA	INF	0.000	**Yes**	0	5
								**No**	18	144
	Cereal	0.991	-16.643	0.000	0.000	INF	0.000	**Yes**	0	19
								**No**	18	130
	Legumes	0.691	-0.264	0.768	0.224	2.100	0.788	**Yes**	3	31
								**No**	15	119
	Wine	0.117	1.128	3.089	0.844	9.522	2.567	**Yes**	3	9
								**No**	15	139
	Beer	0.995	-15.269	0.000	NA	INF	0.000	**Yes**	0	2
								**No**	18	147
	Coffee	0.188	-0.864	0.421	0.124	1.136	0.456	**Yes**	3	47
								**No**	15	99
	Tea	0.757	0.206	1.229	0.448	4.212	1.203	**Yes**	15	118
								**No**	3	29
	Rice	0.820	0.205	1.227	0.235	5.142	1.208	**Yes**	2	22
								**No**	4	54

Table S1. b. Association between food consumption and deoxynivalenol (DON).

	**Food Item**	***p*-Value**	**Coefficient**	**OR**	**5%**	**95%**	**RR**	**DON**		
								**Food**	**Yes**	**No**
Habitual Consumption	Dairy	0.832	0.103	1.109	0.479	2.410	1.029	**Yes**	105	39
								**No**	17	7
	Nuts	0.057	-0.988	0.372	0.147	0.828	0.810	**Yes**	90	39
								**No**	31	5
	Whole cereal	0.174	0.575	1.776	0.906	3.682	1.152	**Yes**	37	9
								**No**	81	35
	Capsicum powder	0.607	-0.207	0.813	0.423	1.604	0.944	**Yes**	28	12
								**No**	89	31
	Legumes	0.887	-0.098	0.907	0.257	2.637	0.975	**Yes**	117	43
								**No**	9	3
	Fresh green chili	0.898	-0.049	0.952	0.513	1.805	0.987	**Yes**	36	14
								**No**	81	30
	Fresh red chili	0.211	-0.506	0.603	0.312	1.186	0.857	**Yes**	23	13
								**No**	91	31
	Maize	0.305	1.075	2.929	0.482	17.842	1.491	**Yes**	41	14
								**No**	2	2
	Beer	0.102	0.691	1.996	1.020	4.133	1.187	**Yes**	39	9
								**No**	76	35
	Wine	0.746	-0.116	0.891	0.492	1.602	0.970	**Yes**	63	24
								**No**	56	19
24h Recall	Capsicum powder	0.478	1.012	2.750	0.202	37.359	1.467	**Yes**	121	44
								**No**	1	1
	Pepper (black and white)	0.745	0.368	1.445	0.277	15.389	1.089	**Yes**	4	1
								**No**	119	43
	Curcumin	0.489	-0.644	0.525	0.114	2.806	0.810	**Yes**	3	2
								**No**	120	42
	Ginger	0.367	0.977	2.655	0.584	27.081	1.207	**Yes**	7	1

								**No**	116	44
	Dried milled maize (chuchoca)	0.997	16.779	19357234.403	0.000	NA	1.367	**Yes**	1	0
								**No**	120	44
	Cooked maize (mote de maíz)	0.474	-1.020	0.361	0.027	4.899	0.680	**Yes**	1	1
								**No**	122	44
	Fresh maize	0.885	0.100	1.105	0.380	3.899	1.026	**Yes**	9	3
								**No**	114	42
	Peanuts	0.375	0.587	1.798	0.660	6.133	1.141	**Yes**	14	3
								**No**	109	42
	Walnuts	0.529	0.506	1.658	0.502	7.757	1.120	**Yes**	9	2
								**No**	114	42
	Other nuts	0.730	0.391	1.479	0.284	15.744	1.096	**Yes**	4	1
								**No**	119	44
	Dried fruit (raisins, peaches, figs)	0.728	-0.308	0.735	0.181	3.747	0.912	**Yes**	4	2
								**No**	117	43
	Dairy	0.015	-0.875	0.417	0.228	0.749	0.793	**Yes**	49	27
								**No**	74	17
	Meat	0.902	-0.045	0.956	0.521	1.728	0.988	**Yes**	78	29
								**No**	45	16
	Salmon	0.489	-0.644	0.525	0.114	2.806	0.810	**Yes**	3	2
								**No**	120	42
	Cereal	0.306	-0.524	0.592	0.259	1.417	0.850	**Yes**	12	7
								**No**	110	38
	Legumes	0.632	0.215	1.240	0.608	2.688	1.056	**Yes**	26	8
								**No**	97	37
	Wine	0.902	0.085	1.088	0.374	3.843	1.022	**Yes**	9	3
								**No**	113	41
	Beer	0.995	16.463	14124549	0.000	NA	1.364	**Yes**	2	0
								**No**	121	44
	Coffee	0.053	0.844	2.326	1.169	4.961	1.212	**Yes**	42	8
								**No**	79	35
	Tea	0.123	-0.807	0.446	0.175	1.001	0.838	**Yes**	94	39
								**No**	27	5
	Rice	0.394	-0.452	0.636	0.267	1.551	0.879	**Yes**	16	8
								**No**	44	14

Table S1. c. Association between food consumption and aflatoxin B1 (AFB1) and aflatoxin M1 (AFM1).

	**Food Item**	***p*-Value**	**Coefficient**	**OR**	**5%**	**95%**	**RR**	**AF**		
								**Food**	**Yes**	**No**
Habitual Consumption	Dairy	0.593	-0.364	0.695	0.244	2.430	0.722	Yes	13	131
								No	3	21
	Nuts	0.858	0.121	1.128	0.402	3.909	1.116	Yes	12	117
								No	3	33
	Whole cereal	0.453	-0.503	0.605	0.175	1.681	0.630	Yes	3	43
								No	12	104
	Capsicum powder	0.167	0.778	2.176	0.835	5.435	2.000	Yes	6	34
								No	9	111
	Legumes	0.992	16.081	9637399.439	0.000	NA	Inf	Yes	16	144
								No	0	12
	Fresh green pepper	0.834	-0.130	0.878	0.293	2.320	0.888	Yes	4	46
								No	10	101
	Fresh red pepper	0.234	0.706	2.025	0.726	5.265	1.883	Yes	5	31
								No	9	113
	Maize	0.995	16.061	9440717.809	0.000	NA	Inf	Yes	6	49
								No	0	4
	Beer	0.638	0.276	1.318	0.477	3.384	1.285	Yes	5	43

								No	9	102
	Wine	0.257	-0.672	0.511	0.183	1.333	0.539	Yes	5	82
								No	8	67
24h Recall	Capsicum powder	0.995	15.255	4219678.025	0.000	NA	Inf	Yes	16	149
								No	0	2
	Pepper (black and white)	0.995	-16.192	0.000	NA	INF	0.000	Yes	0	5
								No	16	146
	Curcumin	0.995	-16.192	0.000	NA	INF	0.000	Yes	0	5
								No	16	146
	Ginger	0.770	0.323	1.381	0.133	6.567	1.333	Yes	1	7
								No	15	145
	Dried milled maize (chuchoca)	0.997	-15.562	0.000	NA	Inf	0.000	Yes	0	1
								No	15	149
	Cooked maize (mote de maíz)	0.109	2.309	10.067	0.728	139.341	5.533	Yes	1	1
								No	15	151
	Fresh maize	0.884	-0.157	0.855	0.084	3.820	0.867	Yes	1	11
								No	15	141
	Peanuts	0.741	0.266	1.305	0.278	4.302	1.269	Yes	2	15
								No	14	137
	Walnuts	0.954	-0.062	0.940	0.092	4.247	0.945	Yes	1	10
								No	15	141
	Other nuts	0.433	0.903	2.467	0.228	13.356	2.173	Yes	1	4
								No	15	148
	Dried fruit (raisins, peaches, figs)	0.559	0.659	1.933	0.182	9.850	1.778	Yes	1	5
								No	15	145
	Dairy	0.705	0.199	1.221	0.509	2.930	1.197	Yes	8	68
								No	8	83
	Meat	0.328	0.588	1.800	0.706	5.291	1.710	Yes	12	95
								No	4	57
	Salmon	0.995	-16.192	0.000	NA	INF	0.000	Yes	0	5
								No	16	146
	Cereal	0.336	0.666	1.947	0.549	5.662	1.798	Yes	3	16
								No	13	135
	Legumes	0.619	0.304	1.356	0.455	3.531	1.314	Yes	4	30
								No	12	122
	Wine	0.874	-0.171	0.842	0.083	3.766	0.856	Yes	1	11
								No	15	139
	Beer	0.996	-15.249	0.000	NA	INF	0.000	Yes	0	2
								No	15	150
	Coffee	0.082	0.926	2.524	1.042	6.121	2.280	Yes	8	42
								No	8	106
	Tea	0.214	-0.720	0.487	0.193	1.336	0.529	Yes	11	122
								No	5	27
	Rice	0.876	-0.113	0.893	0.242	2.783	0.906	Yes	3	21
								No	8	50

Table S2. Calculated regressions of the levels of mycotoxins in urine found and the grams per day consumed by the 172 participants of the study. **Table S2a** Day before consumption (24h recall); **Table S2b** Habitual consumption.

Table S2. a. Calculated regressions of the levels of mycotoxins in urine found and the grams per day consumed by the 172 participants of the study: Day before consumption (24h recall).

Mycotoxin	Food Item	*p*-Value	Coefficient	R2	R2_adj
Aflatoxins (B1 + M1)	powder capsicum	0.0271	0.3365	0.1806	0.1478
	pepper	NA	NA	NA	NA
	curcumin	NA	NA	NA	NA
	ginger	0.7358	0.0000	0.4561	0.3654

	dried maize	NA	NA	NA	NA
	cooked maize	NaN	0.0000	NaN	NaN
	fresh maize	0.4038	0.9167	0.0786	-0.0238
	peanut	0.7305	0.0000	0.5020	0.4688
	walnut	0.0267	57.7778	0.4375	0.3750
	other nuts	0.5101	-13.8889	0.1563	-0.1250
	dried fruit	0.5122	2.6667	0.1143	-0.1071
	dairy	0.6643	0.2224	0.0026	-0.0109
	meat	0.0687	0.2797	0.0312	0.0220
	salmon	NA	NA	NA	NA
	cereal	0.6155	0.0806	0.0152	-0.0428
	legumes	0.4852	-0.3544	0.0153	-0.0154
	wine	0.2228	1.4394	0.1446	0.0590
	beer	NA	NA	NA	NA
	coffee	0.5304	0.4468	0.0083	-0.0124
	tea	0.1391	-1.0093	0.0169	0.0093
	rice	0.8395	0.5682	0.0039	-0.0867
Deoxynivalenol (DON)	powder capsicum	0.3198	0.0007	0.0618	0.0032
	pepper	0.6641	-0.0052	0.1128	-0.3308
	curcumin	0.8086	0.0051	0.0877	-0.8246
	ginger	NaN	0.0000	NaN	NaN
	dried maize	NA	NA	NA	NA
	cooked maize	NA	NA	NA	NA
	fresh maize	0.9231	0.0006	0.0014	-0.1412
	peanut	NaN	0.0000	NaN	NaN
	walnut	0.4744	0.0548	0.0755	-0.0566
	other nuts	0.7415	0.0036	0.0668	-0.3997
	dried fruit	0.6087	-0.0016	0.1531	-0.2703
	dairy	0.7923	0.0004	0.0015	-0.0198
	meat	0.6569	-0.0002	0.0026	-0.0105
	salmon	0.9200	-0.0051	0.0157	-0.9686
	cereal	0.4373	0.0004	0.0614	-0.0324
	legumes	0.4325	-0.0005	0.0259	-0.0147
	wine	0.5393	-0.0018	0.0562	-0.0787
	beer	NaN	-0.3257	1.0000	NaN
	coffee	0.4742	-0.0016	0.0129	-0.0118
	tea	0.8019	0.0004	0.0007	-0.0104
	rice	0.2064	-0.0077	0.1912	0.0901
Zearalenone Metabolites (ZEL)	powder capsicum	0.5878	-0.0124	0.0119	-0.0276
	pepper	0.0718	-0.0179	0.7134	0.6179
	curcumin	NA	NA	NA	NA
	ginger	NA	NA	NA	NA
	dried maize	NA	NA	NA	NA
	cooked maize	NA	NA	NA	NA
	fresh maize	0.4038	0.0331	0.0786	-0.0238
	peanut	0.7277	0.0000	0.5021	0.4689
	walnut	0.7659	0.0856	0.0104	-0.0996
	other nuts	NA	NA	NA	NA
	dried fruit	NA	NA	NA	NA
	dairy	0.5133	0.0012	0.0058	-0.0076
	meat	0.9646	0.0000	0.0000	-0.0095
	salmon	NA	NA	NA	NA
	cereal	NA	NA	NA	NA
	legumes	0.5316	-0.0073	0.0123	-0.0185
	wine	0.4953	-0.0321	0.0477	-0.0475
	beer	NA	NA	NA	NA

	coffee	0.4930	0.0140	0.0098	-0.0108
	tea	0.3388	0.0024	0.0071	-0.0006
	rice	0.8910	0.0001	0.0018	-0.0890

Table S2. b. Calculated regressions of the levels of mycotoxins in urine found and the grams per day consumed by the 172 participants of the study: Habitual consumption.

Mycotoxin	Food Item	*p*-Value	Coefficient	R2	R2_adj
Aflatoxins (B1 + M1)	cereal	0.60804	-2.28231	0.00165	-0.00459
	nuts	0.90093	0.40119	0.00010	-0.00604
	capsicum	0.31978	0.64908	0.00627	-0.00002
	dairy (skim)	0.85144	4.90821	0.00021	-0.00585
	dairy (whole)	0.62426	-11.34668	0.00150	-0.00474
	dairy (all)	0.74895	-9.83268	0.00062	-0.00540
	legumes	0.77473	2.92904	0.00048	-0.00540
	maize	0.83010	68.85379	0.00081	-0.01671
	beer	0.76774	-3.62425	0.00051	-0.00536
	wine	0.44085	-14.84479	0.00350	-0.00236
Deoxynivalenol (DON)	cereal	0.31979	0.01599	0.00853	-0.00001
	nuts	0.00288	0.03242	0.07222	0.06442
	capsicum	0.76400	0.00069	0.00079	-0.00790
	dairy (skim)	0.85713	-0.01672	0.00027	-0.00813
	dairy (whole)	0.68513	0.03600	0.00142	-0.00719
	dairy (all)	0.93966	0.00836	0.00005	-0.00828
	legumes	0.48850	-0.02595	0.00388	-0.00416
	maize	0.58986	-0.12253	0.00715	-0.01707
	beer	0.73809	-0.00666	0.00090	-0.00715
	wine	0.80206	-0.01743	0.00051	-0.00755
Zearalenone Metabolites (ZEL)	cereal	0.01355	0.07109	0.03750	0.03148
	nuts	0.62579	-0.01026	0.00146	-0.00466
	capsicum	0.53782	-0.00268	0.00241	-0.00391
	dairy (skim)	0.28205	-0.19515	0.00701	0.00099
	dairy (whole)	0.51654	0.09988	0.00263	-0.00360
	dairy (all)	0.68328	-0.08692	0.00101	-0.00501
	legumes	0.88469	-0.01028	0.00012	-0.00576
	maize	0.42536	-2.38516	0.01118	-0.00616
	beer	0.68585	-0.03439	0.00096	-0.00491
	wine	0.60678	-0.06871	0.00156	-0.00431

Table S3. Levels in ng/kg bw creatinine adjusted, probable daily intake (PDI) and risk estimation by the Hazard Quotient (HQ) or Margin of Exposure (MoE) of the mycotoxins found in urine of the participants of the study. **Table S3a** shows zearalenone metabolites (α and β zearalenol, ZEL); **Table S3b** shows DON; and **Table S3c** shows aflatoxins.

Table S3. a. Levels in ng/kg bw creatinine adjusted, probable daily intake (PDI) and risk estimation by the Hazard Quotient (HQ) or Margin of Exposure (MoE) of zearalenone metabolites (α and β zearalenol, ZEL).

ID	ZEL ng/mg creat	PDI ng/kg bw/day	HQ
78	3.7	829.2	3.3
241	4.0	537.4	2.1
2	33.6	6340.9	25.4
82	13.4	2472.4	9.9
226	12.0	2648.5	10.6
72	5.1	1037.7	4.2
229	8.5	1574.7	6.3
212	158.7	39810.0	159.2
170	95.8	19164.8	76.7
208	38.9	8207.1	32.8

211	1.9	381.4	1.5
224	33.2	8111.7	32.4
223	16.2	3053.4	12.2
274	17.7	2324.4	9.3
235	6.2	1452.5	5.8
240	8.8	2984.4	11.9
7	28.6	5237.5	21.0
68	8.4	1769.6	7.1

Table S3. b. Levels in ng/kg bw creatinine adjusted, probable daily intake (PDI) and risk estimation by the Hazard Quotient (HQ) or Margin of Exposure (MoE) of Deoxynivalenol (DON).

ID	DON ng/mg creat	PDI ng/kg bw/day	HQ
256	243.4	3448.8	3.4
88	43.6	1787.3	1.8
251	11.1	491.8	0.5
40	54.1	784.9	0.8
131	70.8	936.9	0.9
12	87.1	846.9	0.8
78	31.4	613.0	0.6
10	43.8	399.9	0.4
137	14.3	721.5	0.7
93	87.4	2179.0	2.2
257	65.0	1945.8	1.9
154	25.0	1104.2	1.1
278	31.1	2563.0	2.6
2	68.0	1307.4	1.3
82	19.7	748.2	0.7
270	27.1	1713.0	1.7
192	35.9	1345.1	1.3
15	10.2	474.0	0.5
83	28.2	1598.1	1.6
73	19.3	528.0	0.5
92	57.8	1484.0	1.5
210	19.5	464.1	0.5
180	31.4	791.1	0.8
261	85.1	1981.5	2.0
44	57.9	1987.4	2.0
220	60.8	1800.7	1.8
114	7.2	195.6	0.2
193	68.3	1366.2	1.4
38	91.8	2838.4	2.8
268	30.6	910.2	0.9
196	443.0	17852.4	17.9
226	124.7	3594.0	3.6
95	33.3	1514.3	1.5
263	73.6	1657.1	1.7
141	20.2	596.5	0.6
191	15.8	366.3	0.4
183	41.9	977.1	1.0
237	106.5	2881.5	2.9
49	23.7	698.0	0.7
273	331.1	9235.5	9.2
269	41.7	1056.3	1.1
229	17.8	431.9	0.4
260	101.3	2769.7	2.8
187	36.5	1172.6	1.2

266	7.7	213.6	0.2
222	22.4	628.2	0.6
258	118.9	2923.5	2.9
182	38.9	913.6	0.9
238	44.2	1305.2	1.3
213	42.1	1678.5	1.7
265	40.5	1194.0	1.2
208	71.0	1955.7	2.0
155	80.9	2468.2	2.5
211	89.0	2376.2	2.4
224	74.5	2379.3	2.4
231	38.3	1465.8	1.5
249	27.1	806.7	0.8
20	23.9	1004.2	1.0
262	158.1	4006.2	4.0
275	81.2	2686.2	2.7
176	19.9	475.1	0.5
276	65.2	1846.8	1.8
79	35.1	834.2	0.8
264	73.0	2215.3	2.2
130	22.8	536.2	0.5
277	111.9	2746.6	2.7
147	33.5	823.4	0.8
24	27.8	734.6	0.7
134	63.5	1753.3	1.8
149	27.3	731.1	0.7
272	31.2	1049.6	1.0
207	16.7	327.6	0.3
179	10.4	344.0	0.3
171	9.9	268.9	0.3
274	13.4	403.1	0.4
152	15.9	440.2	0.4
235	145.8	4428.6	4.4
138	79.4	2756.7	2.8
175	90.6	2669.4	2.7
144	32.7	744.4	0.7
94	20.1	436.6	0.4
174	23.9	634.5	0.6
29	61.4	1179.1	1.2
76	14.4	382.6	0.4
228	54.8	1756.1	1.8
236	54.5	2222.4	2.2
132	48.3	1512.1	1.5
189	65.6	2157.9	2.2
215	43.0	1738.2	1.7
123	18.4	508.0	0.5
216	77.1	2148.4	2.1
136	53.9	1415.6	1.4
225	15.3	465.9	0.5
230	18.6	477.6	0.5
128	14.8	433.0	0.4
156	67.1	2247.0	2.2
218	23.2	752.1	0.8
84	80.0	2374.7	2.4
37	66.4	1449.0	1.4
142	216.7	4988.6	5.0

151	14.7	213.6	0.2
143	32.1	816.3	0.8
233	829.2	26495.9	26.5
153	26.7	796.9	0.8
177	70.3	1783.7	1.8
181	49.0	903.8	0.9
200	40.6	1280.3	1.3
7	115.1	2749.9	2.7
184	29.6	614.1	0.6
68	25.1	687.4	0.7
25	21.6	1020.7	1.0
173	24.3	632.3	0.6
234	667.3	17709.7	17.7
119	49.6	1496.1	1.5
33	41.0	932.1	0.9
17	60.1	1880.7	1.9
117	11.8	297.0	0.3
51	93.0	2491.2	2.5

Table S3. c. Levels in ng/kg bw creatinine adjusted, probable daily intake (PDI) and risk estimation by the Hazard Quotient (HQ) or Margin of Exposure (MoE) of Aflatoxin B1 (AFB1) and aflatoxin M1 (AFM1).

ID	Aflatoxins ng/mg creat	PDI ng/kg bw/day	MoE
37	0.09	0.1	4202.0
38	0.28	0.4	943.6
78	0.90	1.3	314.3
87	2.56	2.3	174.2
93	0.27	0.3	1335.9
116	0.67	0.7	547.1
125	0.13	0.2	2373.1
137	0.09	0.1	3550.4
143	0.17	0.2	1905.5
147	0.15	0.2	2275.5
178	0.48	0.6	648.5
207	0.24	0.2	1767.9
218	0.26	0.4	987.9
220	6.58	9.4	42.7
234	0.30	0.4	1046.7
245	0.25	0.3	1349.6

Table S4. LC-MS/MS parameters, equations, and recovery for the detection of targeted mycotoxins. **Table S4a**. Parameters of detection; **Table S4b.** Concentration range and equations of the method; **Table S4c**. Recovery of the method in 4 points.

Table S4. a. Parameters of detection of targeted mycotoxins.

Mycotoxin	Chemical Formula	Molecular Weight (g/mol)	Retention Time (min)	Polarity ESI	E. Interface (kv)	Collision Energy (V)	Precursor Ion (*m*/*z*)	Quantitative Ion (*m*/*z*)
DOM-1	C15H20O5	280.3	7.4	(+)	4.5	-15	281	215
OTA	C20H18O6NCl	403.8	12.1	(+)	4.5	-30	404	239
13C20-OTA	-	423.8	12.1	(+)	3.5	-20	424	250
AFB1	C17H12O6	312.2	10.4	(+)	3.5	-20	313	285
13C17-AFB1		329.1	10.4	(+)	3.5	-20	330	300
AFM1	C17H12O7	328.2	9.1	(+)	3.5	-30	329	229
13C17-AFM1	-	345.3	9.1	(+)	2.0	-30	346	273
13C15-DON	-	311.0	6.1	(+)	3.5	-12	312	263
DON	C15H20O6	296.3	6.1	(+)	3.5	-12	297	249
ZEN	C18H22O5	318.3	12.1	(-)	-3.5	20	317	175

13C18-ZEN	-	336.0	12.1	(-)	-3.5	20	335	290
α- ZEL	C18H26O5	322.4	11.3	(-)	-4.5	20	319	275
ß-ZEL	C18H26O5	322.4	10.8	(-)	-4.5	20	319	275
α OTA	C11H10O5Cl	256.6	12.2	(-)	-3.5	20	255	167

Table S4. b. Concentration range and equations of the method of targeted mycotoxins.

Analyte	Concentration Range (ηg/mL)	Equation of the line	R^2
DON	20.1–250.0	y = 0.0102x+0.1555	0.984
DOM-1	4.2–33.3	y = 0.0989x-0.1142	0.997
AFM1	1.1–5.0	y = 0.1556x+0.4999	0.959
AFB1	0.1–1.3	y = 3.4400x-0.1046	0.991
ß-ZEL	2.3–33.3	y = 0.2749x-0.3758	0.993
α-ZEL	3.7–33.3	y = 0.2782x+0.0502	0.993
ZEN	1.7–13.3	y = 0.4939x+0.1806	0.994
OTA	2.1–20.0	y = 1.8455x-1.5514	0.998
α-OTA	0.8–4.2	y = 2.5915x+0.3402	0.995

Table S4. c. Recovery of the method in 4 points of targeted mycotoxins.

Analyte	Recovery (%)	SD	Relative SD
DON	90.0	8.5	9.5
DOM-1	80.9	10.0	12.4
AFM1	82.8	8.8	10.7
AFB1	86.3	7.6	8.8
ß-ZEL	76.4	2.5	3.3
a-ZEL	84.7	1.2	1.4
ZEN	90.7	3.0	3.3
OTA	94.2	2.7	2.9
α-OTA	64.4	2.5	3.8

SD: Standard Deviation
